# Evaluation of the Farsi‐translated Hemorrhoidal Disease Symptom Score and Short Health Scale questionnaires in patients with hemorrhoid disease: A cross‐sectional study

**DOI:** 10.1002/hsr2.1363

**Published:** 2023-06-23

**Authors:** Seyedeh Parisa Fallah Tafti, Laleh Foroutani, Roxana Safari, Alireza Hadizadeh, Behnam Behboudi, Seyed Mohsen Ahmadi Tafti, Mohammad Reza Keramati, Mohammad Sadegh Fazeli, Amir Keshvari, Alireza Kazemeini

**Affiliations:** ^1^ Department of Surgery, Division of Colorectal Surgery, Colorectal Research Center, Imam Hospital Complex Tehran University of Medical Sciences Tehran Iran; ^2^ Research Center for Advanced Technologies in Cardiovascular Medicine, Cardiovascular Research Institute Tehran University of Medical Sciences Tehran Iran

**Keywords:** hemorrhoidal disease, Hemorrhoidal Disease Symptom Score, Short Health Scale

## Abstract

**Background and Aims:**

The Hemorrhoidal Disease Symptom Score (HDSS) is a tool that is scored based on five main symptoms: pain, bleeding, itching, soiling, and prolapse. Furthermore, the Short Health Scale (SHS) is a measurement tool of subjective health and health‐related quality of life. This study was performed to validate the Farsi‐translated Hemorrhoidal Disease Symptom Score (HDSS), and Scale Short Health Scale adapted for hemorrhoidal disease (SHS‐HD) as a measure of symptom severity in patients with hemorrhoid disease.

**Methods:**

In this study, HDSS and SHS‐HD were translated into Farsi. Participants with confirmed hemorrhoid disease completed the questionnaire. Subsequently, the questionnaire's discriminative validity, convergent validity, reliability, sensitivity, and specificity were evaluated.

**Results:**

Data from 31 patients were analyzed (mean age 39.68; 71% male). The results of the analysis showed good internal consistency as Cronbach's *α* for HDSS and SHS were 0.994 and 0.995 respectively. Spearman's correlation coefficient for the test–retest comparison was 0.986 (*p* < 0.01). The responses demonstrated good convergent validity. Moreover, the comprehension and suitability of each question were rated as excellent (Pearson's correlation coefficient = 0.3).

**Conclusions:**

Our findings revealed that the Farsi translation of the HDSS and SHS‐HD can be a valuable tool for evaluating the symptom severity in patients with hemorrhoid disease.

## 1 INTRODUCTION

Hemorrhoids are defined as enlarged and displaced venous cushions within the anal canal.^1^, ^2^, ^3^ Hemorrhoidal cushions are normal parts of the anal canal's anatomy. They play a significant role in the contraction of the anal canal at rest as they engorge with blood. Various factors, including constipation, prolonged straining, exercise, pregnancy, obesity, aging, genetics, increased intra‐abdominal pressure, valves absence within the hemorrhoidal veins, and nutrition, can lead to pathologic changes in hemorrhoid cushions and induce hemorrhoidal disease.^4^, ^5^ The most common complaints of patients suffering from the hemorrhoidal disease are bleeding, itching, soiling, prolapse, and pain.^6^, ^7^, ^8^, ^9^, ^10^

Since many cases are asymptomatic and many symptomatic cases have a propensity for self‐medication, the actual prevalence of hemorrhoidal disease (HD) remains unknown.^3^, ^11^, ^12^ Nevertheless, the prevalence of hemorrhoids is reported to be 4.4% in the United States, 11% in Spain, 14.4% in Korea, 16% in Italy, and 38.93% in Austria.^9^, ^10^, ^11^, ^12^ Hemorrhoids are most prevalent between the ages of 45 and 65.^9^ Obesity, particularly abdominal obesity, multiple pregnancies, high socioeconomic status, and white race are identified risk factors for this anorectal abnormality.^13^, ^14^ Depending on the clinical manifestations and the grade of prolapse, hemorrhoids can be managed medically or surgically.^10^, ^15^ Although the hemorrhoidal disease is not a life‐threatening condition, the disturbing symptoms can cause physical and psychological strain and impact the patient's quality of life.^6^, ^9^, ^13^, ^16^ Patient‐Reported Outcome Measures (PROMs) can be utilized to quantify the severity of hemorrhoidal disease.^14^ PROMs are patient‐completed questionnaires that demonstrate the outcome or severity of symptoms from the patient's perspective.^11^

Several questionnaires have been designed to assess the severity of hemorrhoids. According to a systematic review conducted in 2020 among five proposed hemorrhoid symptom‐specific questionnaires, the questionnaire created by Rørvik and colleagues had acceptable standards according to the consensus‐based standards for selecting health measurement instruments (COSMIN) criteria.^11^, ^16^, ^17^, ^18^ COSMIN criteria were used to assess the validity, reliability, and responsiveness of the HDSS and SHS scoring systems.^17^ The HDSS is a symptom‐scoring questionnaire based on the five primary symptoms of pain, bleeding, itching, soiling, and prolapse.^17^, ^18^, ^19^, ^20^ There is currently no disease‐specific instrument for assessing health‐related quality of life (HRQoL) in HD. SHS is a subjective health assessment instrument.

SHS is a simplified HRQoL evaluation technique that asks only one question for each of its four aspects: functional status, symptom load, disease‐specific concerns, and overall well‐being. SHS was originally developed for inflammatory bowel disease (IBD) patients; however, an HD‐specific modified version focusing on hemorrhoidal disease (SHS‐HD) was subsequently developed. SHS‐HD is a reliable and responsive assessment instrument for HRQoL, while HDSS is a valid, reliable, and responsive measurement tool for symptoms in HD.^16^ These two grading systems accurately depict a patient's symptoms and their impact on quality of life.^17^, ^20^

Translation of a prefabricated questionnaire can be challenging due to the possibility that linguistic differences and interpretations will compromise the accuracy. To assess the effectiveness, efficiency, and psychometrics of a translated questionnaire, certain statistical methods have been proposed. This study aims to translate the questionnaires to Farsi for the first time and to evaluate the validity and reliability of the Farsi translation of HDSS and SHS‐HD in a colorectal center.

## 2 MATERIALS AND METHODS

This explorative cross‐sectional study translated the English version of the HDSS and SHS‐HD questionnaires to Farsi. Face, content, and construct validities, as well as reliability, were evaluated. HDSS and SHS‐HD are reliable and responsive measuring tools for HD. In this study, both questionnaires were used to provide surgeons with a comprehensive picture of the patient's symptoms and their impact on daily life and well‐being. The HDSS evaluates five symptoms, each of which is scored on a 5‐point scale, yielding a total score between 0 and 20. The SHS‐HD is a 7‐point Likert scale with a total score of between 4 and 28. This study was carried out according to COSMIN criteria.

### 2.1 Translation

After receiving permission from the original author, the questionnaire was independently translated into Farsi by four researchers fluent in both Farsi and English (forward translation). Then, they reviewed the translations to ensure that the concepts remained the same, and all inconsistencies were resolved by discussion to reach a final consensus and prepare the provisional Farsi version. The Farsi version was then translated back into English by an English‐native colleague who was fluent in the Farsi language and was unaware of the English version (backward translation). The back‐translated English version was then compared with the original to ensure no conceptual discrepancies. After the final review, the back‐translated draft was emailed to the original authors for approval. Following confirmation, the Farsi version was checked for the content's simplicity, comprehensibility, and accuracy. No change was made to the final Farsi version due to the suitability of the translated content.

### 2.2 Participants and data gathering

This study examined 53 consecutive patients with hemorrhoids who met the inclusion criteria and were referred to Imam Khomeini Hospital Complex in Tehran in 2020. This research also assessed the validity and psychometric weight of the Farsi translation of HDSS and SHS‐HD. The study's primary findings determined the minimum sample size to be 31 cases.

$$\frac{z^{2}\times \left(1-\frac{\alpha }{2}\right)\times \mathrm{Sen}\times (1-\mathrm{Sen})}{d^{2}\times P}.$$

*N* = 31

*à*:0.05

Sen:72

P:4%

d:8

Fifty‐three adult patients suffering from HD were enrolled in this study. The inclusion criteria for this study were a confirmed diagnosis of hemorrhoids, and the ability to read and write in Farsi. Patients with concurrent conditions such as IBD or cancer were excluded. Patients who required intensive care and surgery, such as a thrombosed or strangulated hemorrhoid, were also excluded.

Goligher's classification was used in grading internal hemorrhoids. No surgical intervention was performed on patients in this study. After informing patients about the aim of this study, their consent was obtained, and they were asked to fill out the questionnaire during a clinical visit. The completed questionnaires were gathered to elucidate the comprehensiveness of the Farsi questionnaire, and then errors were meticulously evaluated. The patients were enrolled in the colorectal clinic of Imam hospital complex from February 2020 to February 2021.

### 2.3 Statistical analysis

IBM SPSS AMOS (v. 26) was used for statistical analysis. *p* < 0.05 were considered statistically significant.

### 2.4 Face validity assessment

Face validity was determined based on the responses of 53 randomly selected patients who completed the questionnaire. The patients were asked to express their opinion about the importance, degree of comprehension, and adequacy of each question on the 5‐point Likert scale. An impact score greater than 1.5 was deemed acceptable.

### 2.5 Content validity assessment

The content validity of the Farsi questionnaire was examined by a panel of specialists, including 20 university faculty members and colorectal surgeons, using a 3‐point Likert scale to judge the simplicity, clarity, relevance, and necessity of each item individually. Furthermore, each item's content validation ratio (CVR) and content validity index (CVI) was calculated using the Lawshe method.

### 2.6 Discriminative validity assessment

The questionnaire's discriminative validity was determined by examining the ability of the Farsi versions of the HDSS and SHS‐HD to distinguish between several groups of patients predicted to differ.

### 2.7 Reliability assessment

Internal consistency was measured using Cronbach's *α*; results greater than 0.7 indicate high internal consistency. The questionnaire was administered to patients 14 days apart. All patients' test–retest reliability was evaluated. The test‐retest reliability of all patients was investigated. The intraclass correlation coefficient (ICC) was used to determine the temporal stability between the first and second responses. An ICC > 0.8 was considered reliable. As this study aimed to evaluate the validity of the translation, responsiveness was not assessed.

## 3 RESULTS

### 3.1 Participants

Fifty‐three patients participated in the study and completed the initial questionnaire. The average age of respondents was 39.72 ± 13.65 years (range: 24–67 years). The participants' average body mass index (BMI) was 26.62 ± 4.736. The male‐to‐female ratio was 2.31, including 37 (69.8%) men and 16 (30.2%) women. The participants' golingher's stages are described in Table 1.

**Table 1:** Frequency and percentage distribution of patients by respective hemorrhoid severity according to clinical evaluations.

Hemorrhoid degree	Frequency	Validity percent
First	42	79.2
Second	3	5.7
Third	3	5.7
Fourth	2	3.8
Total	50	100

### 3.2 Face and content validity

For face validity, 53 randomly selected participants of varying ages and professions were interviewed; each question's degree of comprehension and adequacy was evaluated (Pearson's correlation coefficient = 0.3). The Farsi questionnaire was comprehensive and concise for all patients. Regarding CVR, all criteria gained a high score, and all experts believed that all criteria were essential for patient evaluation (r_s_ = 0.3). The content validity of the Farsi version was deemed good by the experts.

### 3.3 Discriminative validity

The discriminative validity analysis of a single sample *t* test revealed that the HDSS and SHS scores could distinguish between older (mean age of 39.68) and younger participants (*p* value 0.05). However, it was unable to classify participants by gender (*p* = 0.574).

### 3.4 Reliability

To assess the reliability of questionnaires we conducted the survey in two separate sessions; by comparing the results of the test with the retest. The results of the analysis showed good internal consistency as Cronbach's *α* for HDSS and SHS were 0.994 and 0.995, respectively. Interclass Correlation Coefficient (ICC) was calculated to be 0.997 (*p* < 0.000), indicating good agreement between the first and second tests. We used nonparametric analysis to assess the correlation between HDSS and SHS questionnaires which showed low positive correlation (Spearman's rho: 0.296 *p*:0.32). the analysis also compared the correlation between total test scores including overall score, HDSS and SHS and their respective retests. Temporal stability was determined through the statistical calculation of Spearman's Correlation Coefficient (SCC) between the results of tests and retests equal to 0.98, which shows that the Farsi version of the questionnaires has very high repeatability in various locations and periods. Further analysis using Pearson's correlation showed that HDSS, SHS and total scores have high positive correlation. (Tables 2, 3, 4).

**Table 2:** The table demonstrates the correlations between domains of SHS, the data is presented in Pearson's correlation and *p* Values.

	Total score SHS	Concern	Well‐being	Life style
Concern	0.934 (*p* < 0.001)			
Well‐being	0.912 (*p* < 0.001)	0.826 (*p* < 0.001)		
Life style	0.907 (*p* < 0.001)	0.812 (*p* < 0.001)	0.730 (*p* < 0.001)	
Activity	0.884 (*p* < 0.001)	0.745 (*p* < 0.001)	0.822 (*p* < 0.001)	0.836 (*p* < 0.001)

Abbreviation: SHS, Short Health Scale.

**Table 3:** The table demonstrates the correlations between domains of HDSS, the data is presented in Pearson's correlation and *p* Values.

	Pain	Itching	Bleeding	Soiling	Swelling
Itching	0.742 (*p* < 0.001)				
Bleeding	0.156 (*p*: 0.263)	0.251 (*p*:0.069)			
Soiling	0.552 (*p* < 0.001)	0.575 (*p* < 0.001)	0.313 (*p*:0.023)		
Swelling	0.509 (*p* < 0.001)	0.637 (*p* < 0.001)	0.637 (*p* < 0.001)	0.389 (*p*:0.004)	
Total score HDSS	0.763 (*p* < 0.001)	0.854 (*p* < 0.001)	0.854 (*p* < 0.001)	0.700 (*p* < 0.001)	0.815 (*p* < 0.001)

Abbreviation: HDSS, Hemorrhoidal Disease Symptom Score.

**Table 4:** This table demonstrates the correlations between the results of HDSS, SHS, and the combined questionnaires, the data is presented in Pearson's correlation and *p* Values.

	Total score	Total score retest	HDSS test	HDSS retest	SHS test
Total score retest	0.994 (*p* < 0.001)				
HDSS test	0.922 (*p* < 0.001)	0.926 (*p* < 0.001)			
HDSS retest	0.901 (*p* < 0.001)	0.915 (*p* < 0.001)	0.988 (*p* < 0.001)		
SHS test	0.953 (*p* < 0.001)	0.940 (*p* < 0.001)	0.762 (*p* < 0.001)	0.737 (*p* < 0.001)	
SHS retest	0.954 (*p* < 0.001)	0.952 (*p* < 0.001)	0.776 (*p* < 0.001)	0.749 (*p* < 0.001)	0.990 (*p* < 0.001)

Abbreviations: HDSS, Hemorrhoidal Disease Symptom Score; SHS, Short Health Scale.

## 4 DISCUSSION

The present study evaluated the validity and reliability of the first Farsi questionnaire to assess the severity of hemorrhoidal disease and its impact on patient's quality of life. The SHS and HDSS questionnaires were combined to create a 9‐question form that provided an overview of the severity of patient's symptoms and the disease's effect on their quality of life. This information can aid physicians in decision‐making about therapeutic choices.

Cronbach's *α* was calculated to evaluate the internal consistency of the Farsi version of the questionnaires. Cronbach's *α* for the Farsi version of the SHS and HDSS questionnaires was 0.95, indicating a high level of internal consistency. The Cronbach's *α* of the original version of the questionnaires was 0.773.^17^, ^19^

According to the ICC, the external consistency of the Farsi version of the SHS and HDSS questionnaires was 0.997 (*p* < 0.005), indicating that these questionnaires have an almost perfect external consistency and external correlation of their questions. According to the study by Rørvik and colleagues the ICC of the original version of the HDSS and the SHS were 0.822 and 0.763, which agrees with our work.^17^, ^19^

To better understand the validity of the test we conducted retests to evaluate if the test had appropriate reproducibility. As the results from the correlations have high values, it can be concluded that there is a good relationship between test and retest results in the Farsi version of SHS and HDSS questionnaires.

Our questionnaire was able to differentiate between patients of various age groups (*p* < 0.05), demonstrating its discriminative validity. The face and content validity of the questionnaire was adequate as it was assessed for each question separately (Pearson's correlation coefficient = 0.3).

Hemorrhoidal disease classifications are prerequisite for assessment of standard parameters for treatment guidelines, as well as research applications. The most widely used classification criteria is Goligher Classification which categorizes internal haemorrhoids based on the presence and severity of prolapse. The Goligher classification, has a number of drawbacks in respect to symptoms and their effects on quality of life; moreover, the etiopathogenesis of the disease, and certain clinical situations such circumferential prolapse or solitary prolapsed pile are neglected in this scoring system.

Various grading and classifications have been devised to provide a more comprehensive assessment. In a study conducted in 2009, Nystrom and colleagues used a five point questionnaire which includes discomfort, pain, itching, soiling, and manual reducibility. Even though this questionnaire is valid, it fails to assess prolapse. Meanwhile, other grading systems such as HDSS and SHS assess the frequency of symptoms which can help providing a better and more accurate diagnosis. Meanwhile, Another scoring system developed by Sodergren can interpret and identify the most relevant symptoms and severity of diseases. This system can also be used to monitor the progression of disease.^20^, ^21^, ^22^, ^23^, ^24^

HDSS is a symptom‐scoring tool based on five main symptoms (pain, itching, bleeding, soiling, and prolapse). An SHS is a measurement tool of subjective health. SHS is a simplified HRQoL instrument with only one question in its four dimensions: functional status, symptom burden, disease‐specific concerns, and general well‐being. This study aimed to assess the Farsi version of both HDSS and SHS questionnaires.^3^, ^13^, ^14^, ^16^, ^25^, ^26^

At present, Rørvik's HDSS and SHS questionnaires are the most valid tests for diagnosing and evaluating hemorrhoids. Both these scoring systems have been utilized to evaluate outcomes and treatment planning. A recent review article demonstrated that these two scoring systems outperform other scoring systems; consequently, the questionnaire was translated into Farsi. A simple translation of the questionnaire without any modification for cultural and linguistic context can result in an inaccurate measurement. According to our results the farsi translated HDSS and HSS questionaaires show both reliability and accuracy.^7^, ^10^, ^18^, ^26^

In a recent study, the COSMIN criterion was used to evaluate the methodological quality of both HDSS and HSS, which concluded that both tests exhibit acceptable qualities for evaluation and scoring. Future research in this field should include additional tests and evaluations utilizing both COSMIN criteria and a gold standard criterion for hemorrhoidal disease, with both English and Farsi versions.^17^, ^26^, ^27^, ^28^

## 5 CONCLUSION

In conclusion, the Farsi translations of the HDSS and SHS questionnaires are valid and reliable. The translated questionnaires can depict the severity of symptoms and quality of life in patients with hemorrhoidal disease. The original study indicates that the HDSS is reliable and valid, and the SHS is also valid.

## AUTHOR CONTRIBUTIONS

**Seyedeh Parisa Fallah Tafti**: Conceptualization; formal analysis; investigation; writing—original draft; writing—review & editing. **Laleh Foroutani**: Conceptualization; data curation; formal analysis; validation; writing—original draft; writing—review & editing. **Roxana Safari**: Conceptualization; data curation; methodology; visualization; writing—original draft; writing—review & editing. **Alireza Hadizadeh**: Data curation; formal analysis; investigation; writing—original draft; writing—review & editing. **Behnam Behboudi**: Conceptualization; resources; validation; writing—original draft. **Seyed Mohsen Ahmadi Tafti**: Conceptualization; data curation; project administration; resources; software; supervision; validation; visualization; writing—original draft; writing—review & editing. **Mohammad Reza Keramati**: Investigation; visualization; writing—original draft. **Mohammad Sadegh Fazeli**: Software; writing—original draft; writing—review & editing. **Amir Keshvari**: Resources; visualization; writing—original draft. **Alireza Kazemeini**: Data curation; formal analysis; writing—original draft.

## CONFLICT OF INTEREST STATEMENT

The authors declare no conflict of interest.

## ETHICS STATEMENT

This study was approved by the Research Deputy and the Ethics Committee of the Tehran University of Medical Sciences (Reference number: IR.TUMS.MEDICINE.REC.1398.444) and conducted per the ethical standards outlined in the 1964 Declaration of Helsinki and all subsequent revisions. A written informed consent form was obtained from all participants. All authors acknowledge their participation and consent for publication.

## TRANSPARENCY STATEMENT

The lead author Seyed Mohsen Ahmadi Tafti affirms that this manuscript is an honest, accurate, and transparent account of the study being reported; that no important aspects of the study have been omitted; and that any discrepancies from the study as planned (and, if relevant, registered) have been explained.

## Supporting information

Supporting information.

Supporting information.

## Data Availability

Data is available on request due to privacy/ethical restrictions.

## References

[1] Acheson AG , Scholefield JH . Management of haemorrhoids. BMJ. 2008;336(7640):380‐383. 18276714 10.1136/bmj.39465.674745.80 PMC2244760

[2] Kibret AA , Oumer M , Moges AM . Prevalence and associated factors of hemorrhoids among adult patients visiting the surgical outpatient department in the University of Gondar Comprehensive Specialized Hospital, Northwest Ethiopia. PLoS One. 2021;16(4):e0249736. 33878128 10.1371/journal.pone.0249736 PMC8057569

[3] Lohsiriwat V . Hemorrhoids: from basic pathophysiology to clinical management. World J Gastroenterol. 2012;18(17):2009. 22563187 10.3748/wjg.v18.i17.2009 PMC3342598

[4] Sneider EB , Maykel JA . Diagnosis and management of symptomatic hemorrhoids. Surg Clin North Am. 2010;90(1):17‐32. 20109630 10.1016/j.suc.2009.10.005

[5] Agbo S . Surgical management of hemorrhoids. J Surg Tech Case Rep. 2011;3(2):68. 22413048 10.4103/2006-8808.92797 PMC3296437

[6] Lee J‐H , Kim H‐E , Kang J‐H , Shin J‐Y , Song Y‐M . Factors associated with hemorrhoids in Korean adults: Korean national health and nutrition examination survey. Korean J Fam Med. 2014;35(5):227. 25309703 10.4082/kjfm.2014.35.5.227 PMC4192796

[7] Keong SYJ , Tan HK , Lamawansa MD , Allen JC , Low ZL , Østbye T . Improvement in quality of life among Sri Lankan patients with haemorrhoids after invasive treatment: a longitudinal observational study. BJS open. 2021;5(2):zrab014. 33960376 10.1093/bjsopen/zrab014 PMC8088290

[8] Riss S , Weiser FA , Riss T , Schwameis K , Mittlböck M , Stift A . Haemorrhoids and quality of life. Colorectal Dis. 2011;13(4):e48‐e52. 20977590 10.1111/j.1463-1318.2010.02480.x

[9] Riss S , Weiser FA , Schwameis K , et al. The prevalence of hemorrhoids in adults. Int J Colorectal Dis. 2012;27(2):215‐220. 21932016 10.1007/s00384-011-1316-3

[10] Jakubauskas M , Poskus T . Evaluation and management of hemorrhoids. Dis Colon Rectum. 2020;63(4):420‐424. 32132463 10.1097/DCR.0000000000001642

[11] Gram‐Hanssen A , Tolstrup A , Zetner D , Rosenberg J . Patient‐reported outcome measures for patients undergoing inguinal hernia repair. Front Surg. 2020;7:17. 32373624 10.3389/fsurg.2020.00017 PMC7177003

[12] Mott T , Latimer K , Edwards C . Hemorrhoids: diagnosis and treatment options. Am Fam Physician. 2018;97(3):172‐179. 29431977

[13] Lee MJ , Morgan J , Watson AJM , Jones GL , Brown SR . A validated severity score for haemorrhoids as an essential prerequisite for future haemorrhoid trials. Tech Coloproctol. 2019;23(1):33‐41. 30725242 10.1007/s10151-019-01936-9 PMC6394714

[14] Johanson JF , Sonnenberg A . The prevalence of hemorrhoids and chronic constipation. Gastroenterology. 1990;98(2):380‐386. 2295392 10.1016/0016-5085(90)90828-o

[15] Sandler RS , Peery AF . Rethinking what we know about hemorrhoids. Clin Gastroenterol Hepatol. 2019;17(1):8‐15. 29601902 10.1016/j.cgh.2018.03.020 PMC7075634

[16] Tsang S , Royse C , Terkawi A . Guidelines for developing, translating, and validating a questionnaire in perioperative and pain medicine. Saudi J Anaesth. 2017;11(suppl 1):80. 10.4103/sja.SJA_203_17 PMC5463570 28616007

[17] Rørvik HD , Styr K , Ilum L , et al. Hemorrhoidal disease symptom score and short health ScaleHD: new tools to evaluate symptoms and health‐related quality of life in hemorrhoidal disease. Dis Colon Rectum. 2019;62(3):333‐342. 30451751 10.1097/DCR.0000000000001234

[18] Karthikeyan G , Manoor U , Supe S . Translation and validation of the questionnaire on current status of physiotherapy practice in the cancer rehabilitation. J Cancer Res Ther. 2015;11(1):29. 25879332 10.4103/0973-1482.146117

[19] Nyström PO , Qvist N , Raahave D , Lindsey I , Mortensen N . Randomized clinical trial of symptom control after stapled anopexy or diathermy excision for haemorrhoid prolapse. Br J Surg. 2010;97(2):167‐176. 20035531 10.1002/bjs.6804

[20] Gallo G , Martellucci J , Sturiale A , et al. Consensus statement of the Italian society of colorectal surgery (SICCR): management and treatment of hemorrhoidal disease. Tech Coloproctol. 2020;24:145‐164. 31993837 10.1007/s10151-020-02149-1 PMC7005095

[21] Gartell PC , Sheridan RJ , McGinn FP . Out‐patient treatment of haemorrhoids: a randomized clinical trial to compare rubber band ligation with phenol injection. Br J Surg. 1985;72(6):478‐479. 3893619 10.1002/bjs.1800720624

[22] Khan S , Pawlak SE , Eggenberger JC , et al. Surgical treatment of hemorrhoids: prospective, randomized trial comparing closed excisional hemorrhoidectomy and the Harmonic Scalpel® technique of excisional hemorrhoidectomy. Dis Colon Rectum. 2001;44(6):845‐849. 11391146 10.1007/BF02234706

[23] Li Y‐D . Excisional hemorrhoidal surgery and its effect on anal continence. World J Gastroenterol. 2012;18(30):4059. 22912558 10.3748/wjg.v18.i30.4059 PMC3420004

[24] Pucher PH , Qurashi M , Howell AM , et al. Development and validation of a symptom‐based severity score for haemorrhoidal disease: the Sodergren score. Colorectal Dis. 2015;17(7):612‐618. 25603811 10.1111/codi.12903

[25] Sperber AD . Translation and validation of study instruments for cross‐cultural research. Gastroenterology. 2004;126:S124‐S128. 14978648 10.1053/j.gastro.2003.10.016

[26] Jin J , Xia W , Connolly A , Hill AG . Symptom‐based scoring for haemorrhoidal disease: a systematic review. Colorectal Dis. 2020;22(11):1518‐1527. 32639663 10.1111/codi.15253

[27] Sheikh P , Régnier C , Goron F , Salmat G . The prevalence, characteristics and treatment of hemorrhoidal disease: results of an international web‐based survey. J Comp Eff Res. 2020;9(17):1219‐1232. 33079605 10.2217/cer-2020-0159

[28] Mokkink LB , Terwee CB , Knol DL , et al. The COSMIN checklist for evaluating the methodological quality of studies on measurement properties: a clarification of its content. BMC Med Res Methodol. 2010;10(1):22. 20298572 10.1186/1471-2288-10-22 PMC2848183

