# Supplementary figures and images for: Evaluation of the Farsi‐translated Hemorrhoidal Disease Symptom Score and Short Health Scale questionnaires in patients with hemorrhoid disease: A cross‐sectional study
Source: Health Sci Rep. 2023 Jun 23;6(6):e1363. doi: 10.1002/hsr2.1363 (PMC10290184; doi:10.1002/hsr2.1363)

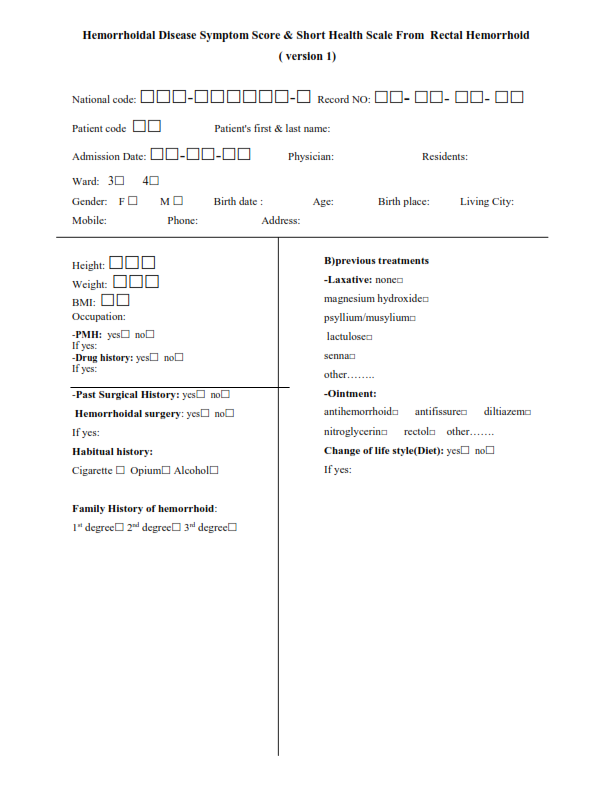

Supplement: Supplementary file 1 — Supporting information. [file HSR2-6-e1363-s002.png]

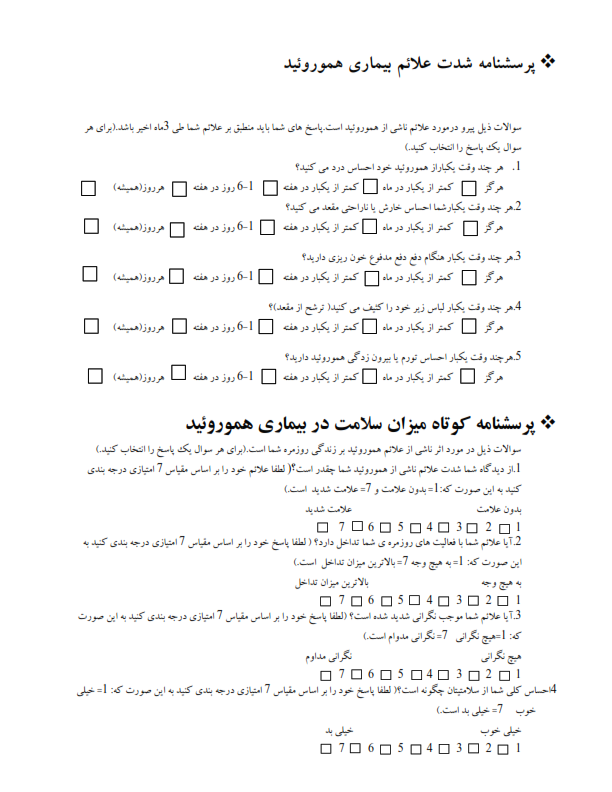

Supplement: Supplementary file 2 — Supporting information. [file HSR2-6-e1363-s001.png]
